# Neural and Molecular Contributions to Pathological Jealousy and a Potential Therapeutic Role for Intranasal Oxytocin

**DOI:** 10.3389/fphar.2021.652473

**Published:** 2021-04-20

**Authors:** Xiaoxiao Zheng, Keith M. Kendrick

**Affiliations:** The Clinical Hospital of Chengdu Brain Science Institute, MOE Key Laboratory for Neuroinformation, University of Electronic Science and Technology of China, Chengdu, China

**Keywords:** pathological jealousy, intranasal oxytocin, partner bonds, social reward, dopamine, serotonin

## Abstract

Romantic jealousy, especially in its pathological form, is a significant contributor to both domestic abuse, including partner sexual coercion and even murder, although relatively little research has been conducted on it. Both obsessive and delusional forms have been identified although only the latter is currently recognized as a pathological disorder. Studies in both clinical and healthy populations have identified altered fronto-striatal responsivity as being associated primarily with romantic jealousy and to date drug based treatments have targeted both dopaminergic and serotonergic systems. However, there is increasing interest in a potential role for the neuropeptide oxytocin, which can also modulate dopaminergic and serotonin systems in the brain and has been shown to altered in other psychotic conditions, such as schizophrenia and obsessive compulsive disorder. Recent studies in healthy populations have reported that when oxytocin is administered intranasally it can influence the brain to promote strengthening of romantic bonds and reduce romantic jealousy in both men and women evoked in either imagined or real contexts. These findings indicate a possible therapeutic use of intranasal oxytocin administration in pathological jealousy.

## Introduction

Jealousy has been defined as “a perception of threat of loss of a valued relationship to a real or imagined rival which includes affective, cognitive and behavioral components” (Mullen, 1991). As such it is a negative emotion involving feelings of resentment, deception, hurt and loss of trust. While jealousy is a widely experienced emotion it is generally considered pathological (morbid) when it goes beyond the level of possessiveness considered acceptable by society (Ecker, 2012). The current review aims to summarize our current understanding of the different forms of pathological romantic jealousy and its neural and neurochemical control and then focusses on the potential for intranasal administration of the neuropeptide oxytocin for reducing it through its actions on strengthening and maintaining romantic bonds and interactions with dopamine and serotonin.

Both obsessive and delusional forms of pathological romantic jealousy are associated with self-harm and predominately male-to-female violence such as domestic abuse and even murder (Camicioli, 2011). A community-based study reported that 15% of men and women had, at some time, been subjected to physical violence by a jealous partner (Mullen and Martin, 1994) and it has been suggested that up to 20% of all murders are contributed to by romantic jealousy (White and Mullen, 1989). Significant associations between jealousy and intimate partner sexual coercion have also been reported in men (Snead and Babcock, 2019). The incidence of pathological romantic jealousy is estimated to be 0.5–1% of the population (Soyka and Schmidt, 2011) although only the delusional form is recognized as a disorder under DSM V as a sub-category of delusional psychosis (APA, 2013). Individuals with obsessive romantic jealousy suffer from unpleasant and irrational jealous ruminations that their partner could be unfaithful and engage compulsive checking of the partner’s behavior, whereas those suffering from delusional jealousy form strong false beliefs that their partner is unfaithful without having any real proof (Batinic et al., 2013). A classic example of delusional romantic jealousy is Shakespeare’s character Othello who constantly believes his wife Desdemona is committing adultery and consumed by jealousy, murders her in a fit of rage before committing suicide. Indeed, delusional jealousy is often referred to as “Othello syndrome”. However, despite the serious impact that pathological jealousy can have on both relationships and society in general we still understand relatively little about its control or effective treatments, so there is an urgent need for research leading toward improved therapeutic options.

While the major focus has been on pathological forms or romantic jealousy, like other disorders, it is reasonable to consider it in a dimensional manner in the same way as other disorders, with jealousy traits being present in everyone and normality compared to pathology only differing in terms of the intensity and irrationality of the feelings experienced. Multidimensional scale questionnaires, such as the Multidimensional Jealousy Scale (Pfeiffer and Wong, 1989), have therefore been developed to span both trait and pathological romantic jealousy. Romantic jealousy is viewed as an evolved complex social emotion characterized by the perceived threat of losing a relationship with a loved one due to a rival and considered to function as a protection from their partial or total loss (Buss and Haselton, 2005). Indeed, mild romantic jealousy can help stabilize relationships by arousing sexual passion and increasing commitment (Buss, 2000). Unlike the basic emotions, including anger, disgust, fear, happiness, sadness, and surprise (Ekman, 1999; Plutchik, 1980), jealousy does not have a distinctive recognizable facial expression and is considered as a compound emotion comprising a mixture of anger, fear, sadness and surprise (Hupka, 1984) serving primarily to help maintain stable relationships and thereby joint parental care. Romantic jealousy generally requires social interactive contexts involving relationship triangles and there are some sex differences with females across cultures more concerned by emotional infidelity but males by sexual infidelity (see Buss et al., 1992; Sagarin et al., 2012; Buss, 2018) in line with the general evolutionary concept of men being more concerned with ensuring their paternity and women with maintaining a stable relationship with a partner to assist in caring for their offspring. This sex difference can even be observed in cases of pathological jealousy with female patients more focused on their partner’s emotional infidelity whereas male patients are more focused on their sexual infidelity (Easton et al., 2007).

### Neural Substrates of Pathological and Trait Romantic Jealousy

A small number of neuroimaging and neuropathology studies in humans, have demonstrated that pathological jealousy is particularly associated with altered fronto-striatal circuitry, the ventral medial prefrontal cortex (vmPFC), thalamus, insula and amygdala. These circuits are involved in the control of a range of behaviors including reward and emotion processing, impulsivity, mentalizing/self-related processing and interoception/salience processing and, most notably, dopaminergic and serotoninergic signaling (see Marazziti et al., 2013; Samad et al., 2019 for reviews). Alterations in the vmPFC and its connections have been proposed to be of particular importance given their involvement in the generation of affective meaning (Roy et al., 2012). In addition to its role in reward processing prefrontal cortex coupling with the basal ganglia also plays an important role in habit formation (Yin and Knowlton, 2006) and thus dorsal striatal and related prefrontal connections and their associated dopaminergic and serotonin signaling may be involved in the progressive transformation of jealousy into a habitual behavior (Marazziti et al., 2013).

A number of case reports have suggested that damage to the right or left frontal lobe is associated with delusional jealousy (see Cipriani et al., 2012) and one study has reported that vmPFC lesions are associated with impaired understanding of envy (Shamay-Tsoory et al., 2007). Interestingly, a study on 105 patients with Othello syndrome has reported that 76.7% had a neurodegenerative disorder with gray matter loss in the frontal and temporal lobes linked to the presence of delusions, and thus pathological jealousy may be contributed to by neurodegenerative changes in the brain (Graff-Radford et al., 2012).

A few neuroimaging studies have investigated the neural basis of jealousy in healthy populations either using exposure to jealousy-evoking contexts or as a function of trait jealousy scores. In male monkeys confronted with threats to their exclusive sexual access to a female partner were associated with increased activity in the amygdala, striatum and superior temporal sulcus, the temporal pole in right hemisphere and bilateral insula (Rilling et al., 2004). In healthy humans evoked jealousy is accompanied by increased activation in the basal ganglia, and frontal lobe, particularly vmPFC, with exaggerated jealousy also being associated with increased interpersonal aggression (Harmon-Jones et al., 2009; Sun et al., 2016). Jealousy evoked in women listening to descriptions of their own experiences of infidelity was also found to produce enhanced activation in brain regions associated with processing different negative emotions, such as the medial frontal cortex, anterior cingulate and insula as well as the fronto-striatal-thalamo-frontal network involved with habit formation and obsessive–compulsive behavior (Steis et al., 2019). One study has also reported sex differences in neural responses during evoked jealousy with men showing greater activation than women in regions involved in sexual and aggressive behaviors, such as the amygdala and hypothalamus, and women in the posterior superior temporal sulcus (Takahashi et al., 2006).

An important issue in determining neural substrates associated with romantic jealousy is that most studies have not controlled for contributions from trait aggression. In a recent study using a dimensional approach with healthy subjects we therefore established associations between neural substrates responding to threatening (angry) faces and trait romantic jealousy while controlling for trait aggression and gender. Our findings revealed that individuals with higher trait romantic jealousy exhibited greater activation in response to angry, but not other emotional faces, the frontal cortex (inferior frontal gyrus) and dorsal striatum as well as the insula, hippocampus, thalamus, fusiform gyrus, superior parietal lobule and bilateral cerebellum. Functional connectivity between the frontal cortex and dorsal striatum was also stronger in more jealous individuals during processing of angry faces (Zheng et al., 2019). This pattern of neural changes is very similar to that reported to show altered responses in individuals with pathological jealousy, underlining the utility of employing a dimensional approach to help establish neural and neurochemical systems involved. While dorsal striatum activation is associated with the receipt of rewards (Delgado, 2007), it also occurs during the processing of negative valence stimuli (Carretié et al., 2009), including viewing those who have rejected individuals romantically (Fisher et al., 2010). Thus, greater activation of the dorsal striatum and its functional connectivity with the frontal cortex may reflect an enhanced responsivity to negative emotional stimuli, particularly those associated with social threat. Interestingly, no associations between romantic jealousy scores and resting state functional connectivity were found suggesting that enhanced jealousy responses may be due to altered dynamic responses to emotional stimuli rather than intrinsic resting state changes (Zheng et al., 2019).

### Current Drug Treatments

In line with evidence from neural studies of romantic jealousy indicating involvement of fronto-striatal and limbic dopaminergic and serotonergic systems (Marazziti et al., 2015; Zheng et al., 2019) drugs targeting both transmitters and their receptors have primarily been used to treat pathological jealousy. Delusional jealousy with its psychotic symptoms tends to be treated with anti-psychotics targeting the dopamine system, such as pimozide (Byrne and Yatham, 1989; Samad et al., 2019). The link with the dopaminergic system has also been emphasized by observations that a significant proportion of patients with psychotic disorders such as schizophrenia exhibit pathological jealousy (Soyka and Schmidt, 2011). Some Parkinson’s disease patients also develop pathological jealousy symptoms while taking dopamine agonist drugs (Poletti et al., 2012). Obsessional jealousy on the other hand tends to be treated with selective serotonin re-uptake inhibitors in view of its similarities with obsessive compulsive disorder and depressive rumination (Stein et al., 1994; Marazziti, 2003; Ecker, 2012), or serotonin agonists (Almeida, 2017). However, these drugs are primarily targeting the symptoms of pathological jealousy rather than the nature of the romantic bond between partners and this has led to consideration of potential therapeutic effects of targeting neuropeptides such as oxytocin which can influence both the formation and maintenance of partner bonds (Kendrick et al., 2017).

### Possible Role for Intranasal Oxytocin in Treating Romantic Jealousy

Over the last few decades there has been increasing interest in the use of intranasal administration of neuroactive peptides and drugs which do not readily cross the blood-brain barrier to deliver them into the brain and thereby influence both brain and behavior (see Erdö et al., 2018). One neuropeptide which has received considerable attention in this respect is oxytocin which has been demonstrated by a large number of studies using animal models to influence social behavior and bonds and with notable effects on fronto-striatal reward and limbic emotional processing networks (see Kendrick et al., 2017). In humans, the effects of administering oxytocin exogenously via an intranasal route in order to activate its widely distributed network of receptors (Quintana et al., 2019) have been widely investigated (Kendrick et al., 2017). The typical single dose applied is 24IU with the intranasal dispensers delivering 4IUs with each 0.1 ml puff. With adult subjects, doses are self-administered and the standard protocol used is to administer 6 alternate puffs to each nostril with each puff separated by 30 s. The general recommendation is to allow 30–45 min for the peptide to reach maximum concentrations in the brain before observing any functional effects (see Guastella et al., 2013).

**FIGURE 1:**
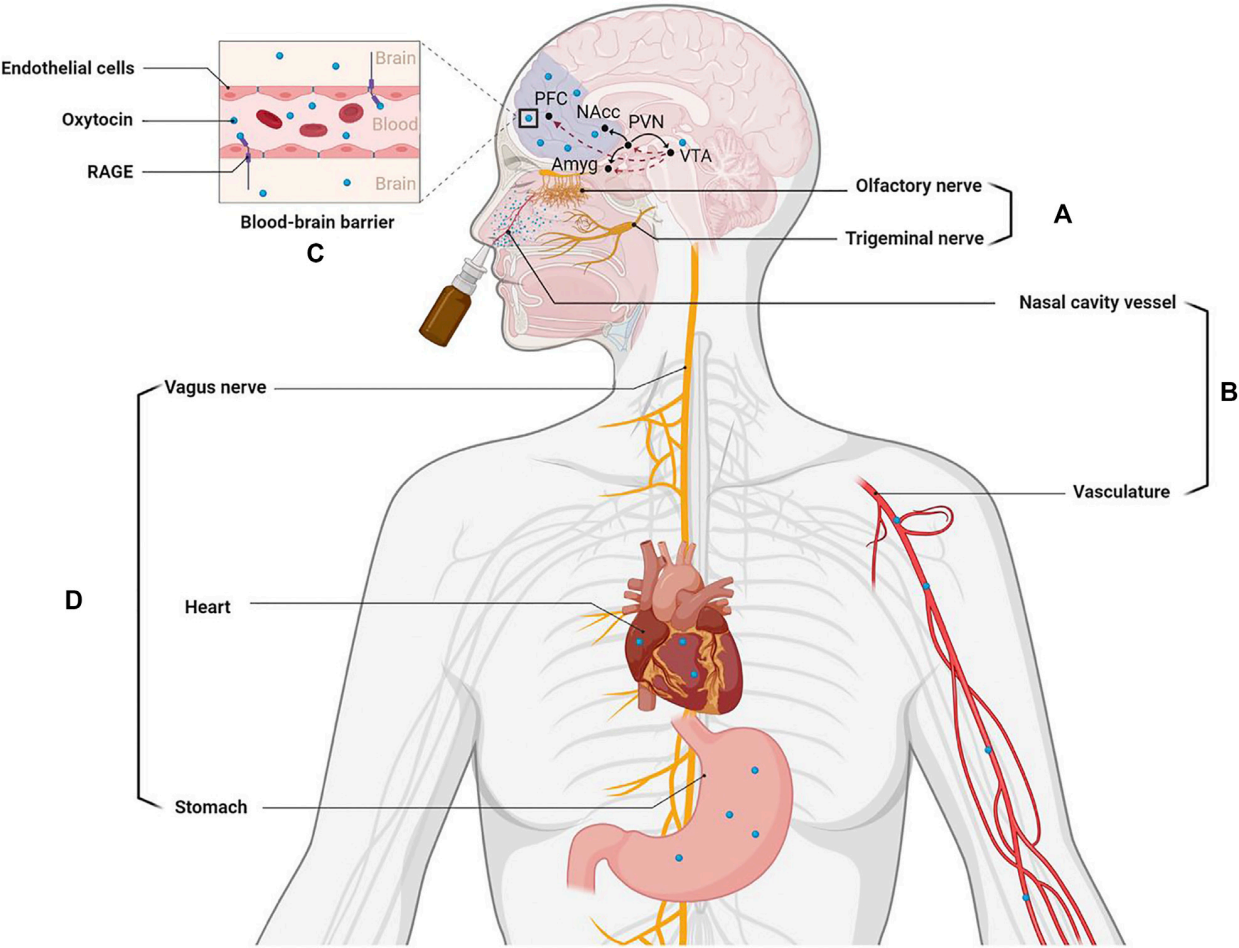
Routes whereby intranasally administered oxytocin can influence brain function and highlighting its influence on the dopaminergic fronto-striatal system implicated in pathological jealousy. **(A)** Nasal spray particles directly enter the brain from the back of nose via olfactory and trigeminal nerves. **(B)** Nasal spray particles are absorbed by nasal cavity blood vessels to enter the peripheral vascular system. **(C)** The oxytocin absorbed into the blood can be transported into the brain via the blood-brain barrier (BBB) by binding to the receptor for advanced glycation end-products (RAGE). **(D)** Intranasally administered oxytocin can also act on its receptors peripherally in the heart and gastrointestinal system to influence brain activity via the vagus nerve. Pathways in the brain: oxytocin indicated by black lines and dopamine by dashed red lines. *PFC*: prefrontal cortex; *NAcc*: nucleus accumbens; *PVN*: paraventricular; *VTA*: ventral tegmental area; *Amyg*: amygdala. Created with BioRender.com.

While the mechanism(s) whereby oxytocin may stimulate its receptors in the brain following intranasal administration has been the subject of some controversy (Leng and Ludwig, 2016) there is now increasing evidence from both animal and human studies that it can potentially exert effects on its receptors in the brain via different routes (see Figure 1). Firstly, there is increasing support for it being able to enter the brain directly from the back of the nose either via olfactory and trigeminal nerves. Secondly, intranasal administered oxytocin is absorbed by blood vessels in the nose and this results in a marked increase in peripheral concentrations in the general circulation. While the blood-brain-barrier is relatively impermeable to oxytocin recent studies have now shown that after binding to the receptor for advanced glycation end-products (RAGE) it can cross it and diffuse into the brain to act on its receptors (Yamamoto and Higashida, 2020). Intranasal oxytocin increases concentrations in the cerebrospinal fluid (Striepens et al., 2013) and can produce altered neural activity and functional effects via its receptors by both of these routes, although there may be route-dependent regional and functional effects (Ferris et al., 2015; Quintana et al., 2016; Dumais et al., 2017; Martins et al., 2020; Kou et al., 2021). It is unclear whether oxytocin entering the brain following intranasal administration targets its receptors in a paracrine manner via circulation in the cerebroventricular system and/or via neural projections from the hypothalamic paraventricular nucleus following stimulation of its autoreceptors. However intranasal administration does lead to widespread activity changes in regions known to contain oxytocin receptors (Martins et al., 2020). Additionally, oxytocin administered intranasally may act on its receptors peripherally in the heart and gastrointestinal system to produce vagally mediated effects on brain function. In this case, vagal stimulation may also be mediated via increased concentrations in peripheral blood or potentially via the peptide leaking down into the mouth and being ingested into the gastrointestinal system (see review by Quintana et al., 2020).

In terms of the effects of intranasal administration of oxytocin which are of relevance to a potential role in modulating romantic jealousy several studies have reported that it can act to strengthen romantic bonds in humans. In both men and women in established relationships intranasal oxytocin can enhance the perceived attractiveness of the face of an existing partner, but not of others, and this is associated with enhanced activation in the striatum and ventral tegmentum regions of the reward system (Scheele et al., 2013; 2016). In another context, intranasal oxytocin also influences men in a relationship to keep a greater distance between them and an attractive female stranger and reduces their interest in approaching erotic pictures of strange females (Scheele et al., 2012). This could be interpreted as oxytocin helping to maintain existing relationships by reducing romantic attraction toward others. Other related findings have demonstrated that intranasal oxytocin can more generally enhance affiliative motivation and recognition of positive valence social stimuli (Zhang et al., 2020) and responses to positive sex and relationship words (Unkelbach et al., 2008). Interestingly, intranasal oxytocin has also been shown to produce a number of sex-dependent effects on perceived attractiveness of individuals and associated neural responses (Ditzen et al., 2013; Gao et al., 2016; Xu et al., 2019). One study reported that in women oxytocin increased, the attractiveness of men with a previous history of emotional and sexual fidelity but in men it increased the attractiveness of previously unfaithful women for having short-term relationships (Xu et al., 2019). On the other hand, intranasal oxytocin has been shown to promote relationship repairing responses more in men than in women (Ditzen et al., 2013; Xu et al., 2017). Importantly a large number of studies have demonstrated that intranasal oxytocin influences the responses and functional connectivity of both cortical and subcortical regions implicated in pathological jealousy, notably the frontal cortex, basal ganglia, insula and parietal and temporal regions (see Kendrick et al., 2017; Jiang et al., 2021). Furthermore, a number of studies have demonstrated that oxytocin can reduce limbic and brainstem responses to threatening face expressions in men (Kirsch et al., 2005; Quintana et al., 2016; Luo et al., 2017; Spengler et al., 2017; Kou et al., 2020), which could be of importance given our finding in healthy subjects that higher trait jealousy is associated with enhanced responses to angry faces in these and other regions (Zheng et al., 2019).

While some studies have reported that intranasal oxytocin can increase envy in non-romantic contexts (Shamay-Tsoory et al., 2009) and reduce forgiveness of trust betrayal in women but not men (Yao et al., 2014), these are in monetary gain/loss rather than relationship contexts. Two more recent studies have both demonstrated that intranasal can reduce jealousy ratings and/or arousal ratings in response to imagined emotional or sexual infidelity by a heterosexual partner (Preckel et al., 2015; Zheng et al., 2021) and also in the context where jealousy is evoked by experiencing being excluded by a partner in an adaptation of the Cyberball game and playing instead with an attractive opposite sex stranger (Zheng et al., 2021). Importantly, the Zheng et al. study found that neither relationship duration nor trust in the partner were associated with jealousy ratings.

Finally, given that a number of studies have associated romantic jealousy with an anxious-ambivalent attachment pattern (Costa et al., 2015), it is interesting that a recent study has demonstrated that a two-week treatment with daily intranasal oxytocin particularly increased attachment security in such insecure attachment individuals (Bernaerts et al., 2017).

Oxytocin has also been shown to be potent modulator in both dopaminergic and serotonergic systems (Kendrick, 2000). In animal models the effects of oxytocin on promoting formation of partner bonds involves it’s interaction with dopaminergic signaling in the striatum (Liu and Wang, 2003) and also oxytocin’s effects enhancing social reward (Dölen et al., 2013) and reducing anxiety (Yoshida et al., 2009) have also been demonstrated to involve interactions with the serotonin system. In the monkey hypothalamus there is also evidence for interactions between oxytocin containing neurons and fibers containing the serotonin transporter (Emiliano et al., 2007). In human males, intranasal oxytocin reduces medial prefrontal cortex responses to the faces of women and also dopamine D2 receptors, although D2 receptor binding was not altered in the striatum (Striepens et al., 2014). Intranasal oxytocin has also been reported to increase levels of 5HT-1A receptors (Mottolese et al., 2014).

While no studies have directly investigated possible associations between pathological jealousy and oxytocin, methylation of the oxytocin receptor is reported to be reduced in schizophrenia and other psychotic disorders (Grove et al., 2016) and obsessive compulsive disorder (Park et al., 2020; Schiele et al., 2020). Altered blood or cerebrospinal fluid concentrations of the peptide have also been reported in both schizophrenia (Rich and Caldwell, 2015) and obsessive compulsive disorder (Marazziti et al., 2015). Additionally, schizophrenia patients with higher blood concentrations of oxytocin exhibit fewer psychosis and social problems (Rubin et al., 2010). Furthermore, intranasal oxytocin has been demonstrated to decrease positive (i.e. psychosis) symptoms in schizophrenia (see Bradley and Woolley, 2017) although there is less evidence for beneficial effects in obsessive compulsive disorder (see Bakermans-Kranenburg and van IJzendoorn, 2013). Importantly, intranasal oxytocin treatments are not associated with any adverse side effects and clinical trials involving chronic treatments lasting up to 3 months in both autism and schizophrenia patients have not reported any significant side effects other than increased urination in young children (MacDonald et al., 2011; Modabbernia et al., 2013; Yatawara et al., 2016). Thus there is considerable support for the potential use of intranasal oxytocin as a potential and safe intervention in the context of pathological jealousy.

## Conclusions and Future Directions

While we are gaining a better understanding of the behavioral, neural and neurochemical systems involved in trait and pathological romantic jealousy there is clearly an urgent need for more research in this field. Current drug treatments targeting either dopaminergic or serotonin systems have produced some success in controlling jealousy behaviors targeting other interacting systems such as the neuropeptide oxytocin may offer a different strategy to strengthen romantic bonds between partners and promote greater tolerance of either real or imagined infidelities. Intranasal administration of oxytocin provides a safe and effective way to target its receptors in the brain and there is increasing evidence that this facilitates reductions in jealousy and can help to strengthen and maintenance of romantic bonds. As yet however, no trials have investigated the potential therapeutic efficacy of oxytocin administration on pathological jealousy, although importantly not only can it reduce trait romantic jealousy but also modulates both dopaminergic and serotonergic systems which are the current main therapeutic drug targets for this disorder.
